# Cytochrome C oxidase Inhibition and Cold Plasma-derived Oxidants Synergize in Melanoma Cell Death Induction

**DOI:** 10.1038/s41598-018-31031-2

**Published:** 2018-08-24

**Authors:** Rajesh Kumar Gandhirajan, Katrin Rödder, Yana Bodnar, Gabriella Pasqual-Melo, Steffen Emmert, Corinne E. Griguer, Klaus-Dieter Weltmann, Sander Bekeschus

**Affiliations:** 1 Leibniz-Institute for Plasma Science and Technology (INP Greifswald), ZIK plasmatis, Felix-Hausdorff-Str. 2, 17489 Greifswald, Germany; 2 University Medical Center Rostock, Clinic for Dermatology and Venerology, Strempelstr. 13, 18057 Rostock, Germany; 3 University of Birmingham, Department of Neurosurgery, 1670 University Boulevard, Birmingham, Alabama 35233 USA

## Abstract

Despite striking advances in the treatment of metastasized melanoma, the disease is often still fatal. Attention is therefore paid towards combinational regimens. Oxidants endogenously produced in mitochondria are currently targeted in pre-clinical and clinical studies. Cytotoxic synergism of mitochondrial cytochrome c oxidase (CcO) inhibition in conjunction with addition of exogenous oxidants in 2D and 3D melanoma cell culture models were examined. Murine (B16) and human SK-MEL-28 melanoma cells exposed to low-dose CcO inhibitors (potassium cyanide or sodium azide) or exogenous oxidants alone were non-toxic. However, we identified a potent cytotoxic synergism upon CcO inhibition and plasma-derived oxidants that led to rapid onset of caspase-independent melanoma cell death. This was mediated by mitochondrial dysfunction induced by superoxide elevation and ATP depletion. This observation was validated by siRNA-mediated knockdown of COX4I1 in SK-MEL-28 cells with cytotoxicity in the presence of exogenous oxidants. Similar effects were obtained with ADDA 5, a recently identified specific inhibitor of CcO activity showing low toxicity *in vivo*. Human keratinocytes were not affected by this combinational treatment, suggesting selective effects on melanoma cells. Hence, targeting mitochondrial CcO activity in conjunction with exogenous pro oxidant therapies may constitute a new and effective melanoma treatment modality.

## Introduction

Cutaneous melanoma is the most deadly form of common skin cancers with a steeply increased incidence over the past three decades^1^. Despite advanced knowledge in the pathogenesis of melanoma, prognosis in advanced stages remains poor^2^. Reactive oxygen species (ROS) may be drivers of carcinogenesis and have thus been long viewed as generally detrimental^3^. Yet, clinical studies failed to show beneficial effects of antioxidants in cancer initiation and progression, with some tumors even progressing under such supplementation^4^. Vice versa it was shown that oxidative stress inhibits and antioxidants increase metastasis of melanoma cells *in vivo*^5–7^. Consequently, there may be a role of ROS as tumor suppressors^8^, and addition of exogenous ROS was shown to be selectively detrimental in many cancer cells^9^.

ROS (Reactive Oxygen Species) are critical second messengers essential for multiple cellular functions^10^. Melanoma cells exhibit high levels of such reactive species and often reduced expression of catalase, glutathione-S-transferase, or manganese-dependent superoxide dismutase (MnSOD) activity^11^. This aberrant redox state is thought to activate redox-sensitive transcription factors and gene expressions leading to cell proliferation, metastasis, and chemo resistance. A majority of endogenous ROS derives from mitochondria^12^, especially from complexes I and III in the electron transport chain governing multiple cellular redox states^13^, membrane potentials^14^, and apoptosis^15^.

It has been previously shown that inhibition of mitochondrial complexes has anti-proliferative effects on tumor cells^16,17^. Even more, mitochondrial priming dictates clinical responses to chemotherapy in cancer patients^18^. In contrast to the Warburg hypothesis^19^, melanoma cells exhibit an increased dependency on oxidative phosphorylation (OXPHOS)^20,21^. This is further confirmed by the fact that ρ0 melanoma cells do not form xenografts^22^ and that non-glycolytic metabolic sources like the Krebs cycle are more active in melanoma cells compared with normal melanocytes^23^. Therefore, targeting the mitochondrial OXPHOS pathway may represent an innovative approach to sensitize melanoma cells for a better efficacy of exogenous therapies like chemotherapy.

We investigated, whether combination of mitochondrial cytochrome C oxidase (CcO) inhibitor and addition of exogenous oxidants exhibits potentiated cytotoxic responses in melanoma cells. To generate oxidants, cold physical plasma was used. Cold plasma is a partially ionized gas expelling a wealth of reactive oxygen and nitrogen species^24^ that are being deposited in cell culture media^25^. Such oxidant-enriched media selectively kills cancer cells *in vitro* by targeting the mitochondrial network^26,27^. We assessed synergistic effects of CcO inhibition and plasma-treated medium in murine B16F0 (non-metastatic), B16F10 (metastatic)^28,29^, and human SK-MEL-28 (BRAF^+^) melanoma cells as well non-malignant human HaCaT keratinocytes. Our results demonstrate a pronounced additive effect of CcO inhibition and oxidants selectively in melanoma cell killing.

## Materials and Methods

### Cell culture

B16F0, B16F10, and SK-MEL-28 melanoma cells as well as non-malignant human HaCaT keratinocytes were cultured in high glucose Dulbecco Minimum Essential Media (DMEM; Invitrogen) supplemented with 10% fetal calf serum (FCS). Cells were incubated with indicated concentrations of potassium cyanide (KCN) or sodium azide (NaN_3_) (Sigma-Aldrich) in Roswell Park Memorial Institute 1640 (RPMI-1640; Invitrogen) media with 1% FCS. In some experiments, cells were incubated with catalase (Sigma-Aldrich). The CcO inhibitor 1-[2-(1-adamantyl) ethoxy]-3-(3,4-dihydro-2(1 H)-isoquinolinyl)-2-propanol hydrochloride] (ADDA 5) was used as a specific inhibitor of COX4 was a kind gift from Prof. Corinne E. Griguer (University of Birmingham, USA). For 2D culture assays, 1 × 10^4^ cells/well were incubated in 96 well culture plates (Nunc) in complete cell culture medium 16 h prior to their experimental use.

### Plasma-Treated Media (PTM)

Plasma-Treated Media (PTM) was generated using the atmospheric pressure argon plasma jet kINPen. The jet is accredited as a medical device for wound treatment in Germany and lacks genotoxic or mutagenic action^30–32^. It was operated at a frequency of 1 MHz with 3 l/min argon gas (99.9999%; Air Liquid) to treat 2 ml of RPMI-1640 media with 1% fetal calf serum (FCS) for 120 s. PTM was immediately used for experiments. The total concentration of H_2_O_2_ in PTM was determined using amplex ultra red reagent (Thermo scientific) according to the recommended protocol. Argon gas-treated medium (with plasma off) served as control throughout all experiments.

### Metabolic activity and cell viability

1 × 10^4^ cells were challenged with ADDA5, KCN or NaN_3_ in the presence or absence of PTM for 3 and 24 hours. Subsequently, wells were loaded with 100 µM of resazurin (Alfa Aesar) that is transformed to fluorescent resorufin by metabolically active cells. The plate was incubated for 2 h at 37 °C. Fluorescence was measured in multimode plate reader (Tecan) at λ_ex_ 535 nm and λ_em_ 590 nm and normalized to untreated control. Four hours after plasma treatment, apoptosis was assessed by staining with cell event™ caspase 3/7 (ThermoFisher) for 30 min at 37 °C. Subsequently, cells were detached using accutase (BioLegend), and accutase containing 4′,6-diamidino-2-phenylindole (DAPI; BioLegend) was added to label terminally dead cells. Cells were subjected to flow cytometric analysis (CytoFlex; Beckman-Coulter). At least 3000 cells were acquired in the caspase^−^/DAPI^−^ gating region. Data analysis was performed utilizing *Kaluza* 1.5a software (Beckman-Coulter).

### Live cell imaging

Cells were challenged with ADDA5, KCN or NaN_3_ in the presence or absence of PTM for 3 h or 24 h. Cells were loaded with either cell death indicator SYTOX Green (1 µM; Thermo scientific), mitochondrial membrane potential indicator, Tetramethylrhodamine ethyl ester (TMRE, 100 nM; AAT bioquest), or superoxide sensitive dye dihydroethidium (DHE, 500 nM, Enzo life sciences) for 30 min at 37 °C. Cells were imaged with a 20X objective using a live cell high throughput imaging system (Operetta CLS; Perkin Elmer) and cell-based quantification was performed with minimum of 300 cells for each condition using dedicated imaging software (Harmony 4.6; Perkin Elmer).

### Small Interfering RNA-Mediated Knockdown of COX4

1 × 10^4^ of SK-MEL-28 cells were transfected with esiRNA against human COX4I1 (Sigma-Aldrich) or non-targeting control esiRNA (Luc) using X-tremeGENE siRNA reagent (Sigma-Aldrich) according to manufacturer’s recommendation. Cells were lysed after 48 h and the knockdown efficiency of CcO confirmed by immunoblotting. The remaining cells were incubated with PTM for 6 h along with respective controls and viability was measured using sytox green staining.

### Immunoblotting

Cells were harvested in ice cold PBS and lysed in RIPA buffer (Pierce) supplemented with complete protease and phosphatase inhibitors (PIM complete; Roche) for 20 min on ice. After centrifugation at 15,000 g for 15 min at 4 °C, total protein in the cell extracts was quantified using Rotiquant (Carl Roth). Forty micrograms of protein was resolved by SDS-PAGE (Invitrogen) and blotted on PVDF membranes (Invitrogen). The membranes were probed with anti-caspase 3, anti-beta actin (Cell Signaling), or anti COX4 (Santa cruz) primary antibodies followed by secondary horse-radish peroxidase-coupled antibodies (Rockland Immunochemicals), and signals were acquired in a chemiluminescence detection system (Applied Biosystems) in a linear dynamic range.

### ATP assay

Cells were treated with KCN or NAN_3_ in the presence or absence of PTM for 2 hours. Total cellular ATP levels quantified using luminescence-based ATP determination kit (Enzo Life Sciences) according to manufactures’ instructions.

### Spheroid assay

SK-MEL-28 cells (5 × 10^3^) were incubated in 96 well ultra-low affinity plates (Perkin Elmer). Seventy-two hours later, cells were challenged with ADDA 5 (1 µM) in the presence or absence of PTM for 24 h. Spheroids were loaded with Sytox green (5 µM; Thermo scientific) and Hoechst 33342 (40 µM; Thermo scientific) for 1 h at 37 °C. Spheroids were imaged with a 5X objective at 50 stacks per well with a live cell high throughput imaging system (Operetta CLS; Perkin Elmer) and quantified by taking the ratio between sytox green and Hoechst 33342 mean fluorescence intensity using dedicated imaging software (Harmony 4.5; Perkin Elmer).

### Statistics

Graphing and statistical analysis was performed using prism 7.03 (GraphPad software). Mean and standard errors were calculated and analyzed according to *t* test or one-way analysis of variances (anova). Levels of significance were indicated as follows: α = 0.05 (*), α = 0.001 (**), α = 0.001 (***).

## Results

### PTM only modestly decreased metabolic activity and cell viability

**Figure 1:**
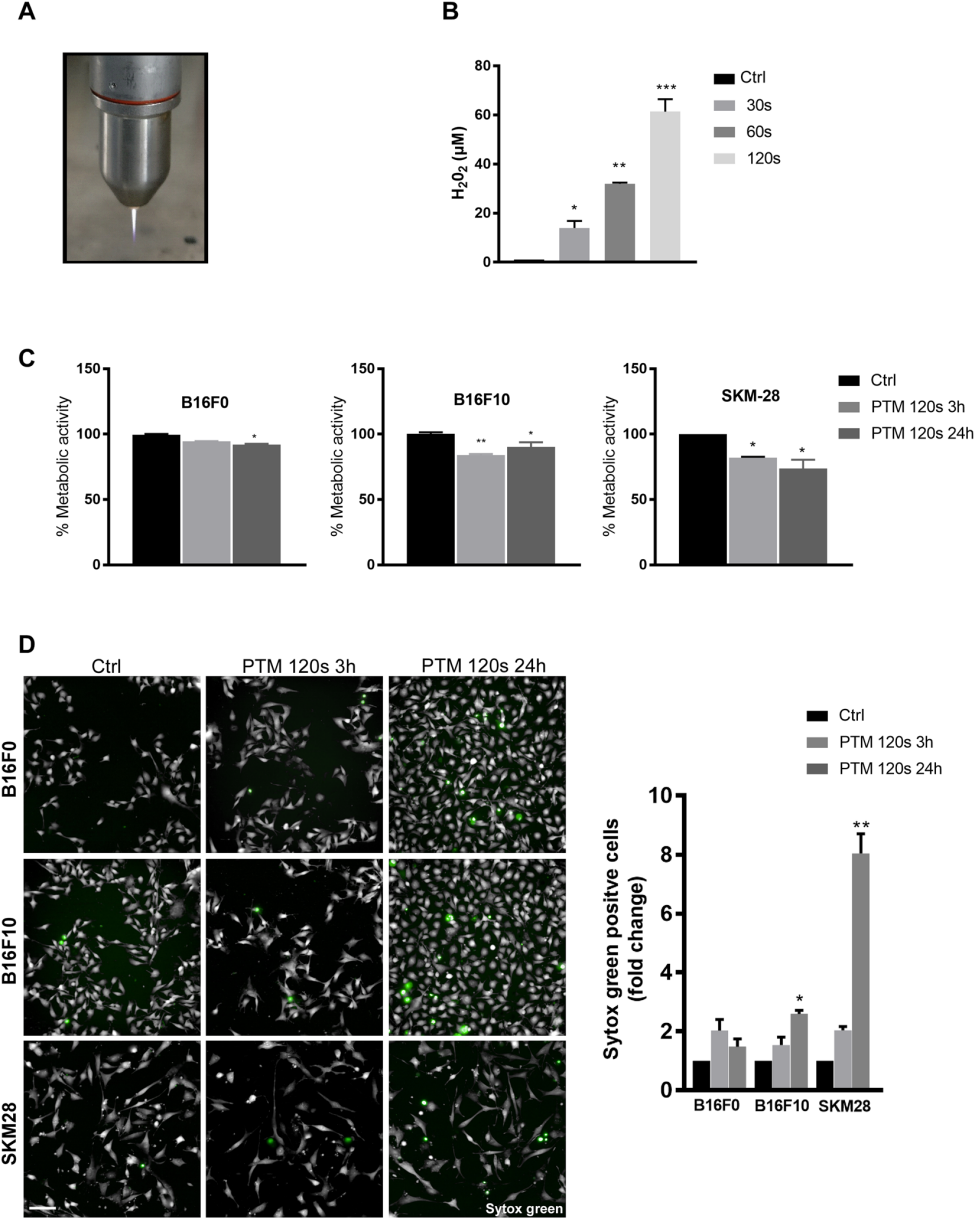
The selected plasma treatment regimen modestly decreases melanoma cell viability. (**A**) Representative image of cold physical plasma jet kINPen™ (**B**) Concentration of H_2_O_2_ following 30, 60, and 120 s of plasma exposure in PBS. Argon exposure for 120 s served as negative control. (**C**) Metabolic activity of B16F0, B16F10, and SK-MEL-28 cells upon incubation in PTM (120 s) for 3 and 24 h, respectively. (**D**) Cytotoxicity in B16F0, B16F10, and SK-MEL-28 cells upon incubation in PTM (120 s) for 3 and 24 h using sytox green to mark terminally dead cells. Scale bar: 100 µm. Data are mean + SEM from three independent experiments.

Hydrogen peroxide (H_2_O_2_) is the most stable oxidant derived from plasma-treated medium (PTM) in this setting and with this device. To ensure a scalability of PTM, we quantified H_2_O_2_ and found a linear increase with treatment time (Fig. 1B). Following optimization on cell lines (data not shown), we chose the 120 s treatment time for subsequent experiments. There was a modest decrease in metabolic activity of all three melanoma cell lines at 3 h (Fig. 1C). However, B16F10 but not B16F0 and SK-MEL-28 cells recovered from the initial insult after 24 h (Fig. 1C). We further enumerated cytotoxic effects of PTM by live cell imaging at 3 h and 24 h post incubation. There was a minor increase in cell death (<10%) in all melanoma cells treated with PTM at both time points (Fig. 1D). Furthermore, SK-MEL-28 were more sensitive to H_2_O_2_ than non-malignant human HaCaT keratinocytes (Fig. S1). Altogether, the application of PTM was of minor toxicity in melanoma cells.

### CcO inhibition sensitized melanoma cells but not keratinocytes to PTM-mediated cell death

**Figure 2:**
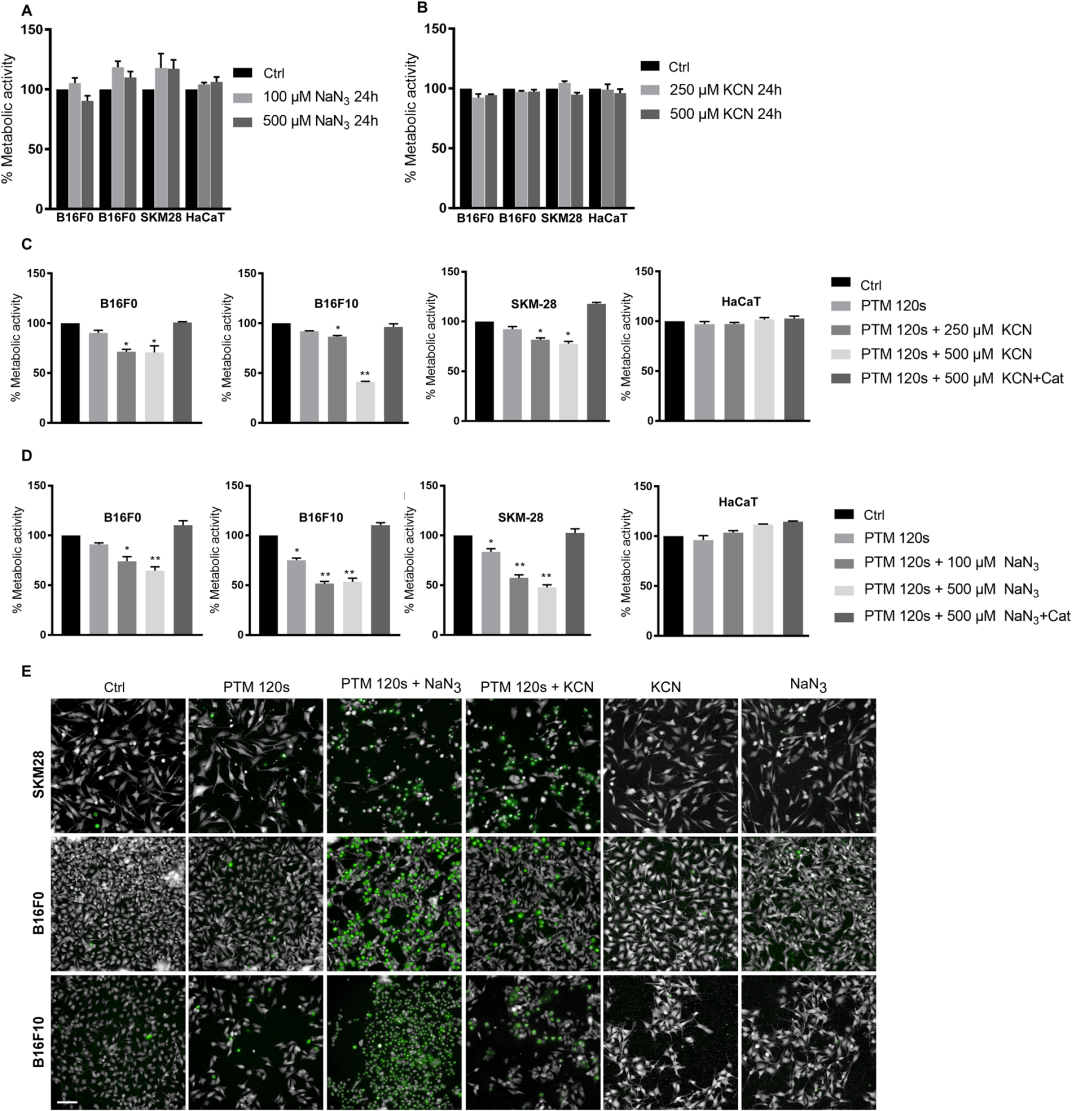
Mitochondrial CcO inhibitors potassium cyanide (KCN) and sodium azide (NaN_3_) sensitize PTM mediated melanoma cell death. (**A**,**B**) Metabolic activity of melanoma cell lines and HaCaT cells upon incubation with mitochondrial CcO inhibitors (KCN or NaN_3_) for 24 h. (**C**,**D**) Metabolic activity of melanoma cell lines and HaCaT cells upon incubation with mitochondrial CcO inhibitors KCN (**C**) and NaN_3_ (**D**) in the presence of PTM for 24 h. (**E**) Cytotoxicity of B16F0, B16F10, and SK-MEL-28 cells upon incubation with mitochondrial CcO inhibitors in PTM (120 s) for 6 h using sytox green assay. Scale bar: 100 µm Data are mean + SEM from three independent experiments.

KCN and NaN_3_ are routinely used biochemical inhibitors of mitochondrial complex IV. We tested these compounds’ ability to induce cytotoxicity in the 3 melanoma cells and non-malignant human keratinocytes (HaCaT). There was no significant reduction in metabolic activity following incubation with KCN (250 or 500 µM) and NaN_3_ (100 or 500 µM) at 24 h (Fig. 2A,B). However, there was a significant reduction of metabolic activity in melanoma cells but not keratinocytes upon co-incubation of cells with CcO inhibitors and PTM (Fig. 2C,D). The observed synergistic effect was also evident by sytox green staining revealing massive induction of cell death following 6 h of treatment (Fig. 2E) and complete cell killing at 24 h (data not shown). Treating cells with KCN (500 µM) or NaN_3_ (500 µM) alone did not induce any cytotoxicity (Fig. 2E). Cell death was rescued by pretreating cells with catalase indicating H_2_O_2_ as primary mediator in the observed cellular toxicity. To confirm a prime role of H_2_O_2_, we repeated the metabolic assays with 50 µM of H_2_O_2_ in the presence of KCN or NaN_3_. (Fig. S2A,B). Again, the effect could be quenched by catalase.

### Synergistic cell death was mediated by mitochondrial dysfunction

**Figure 3:**
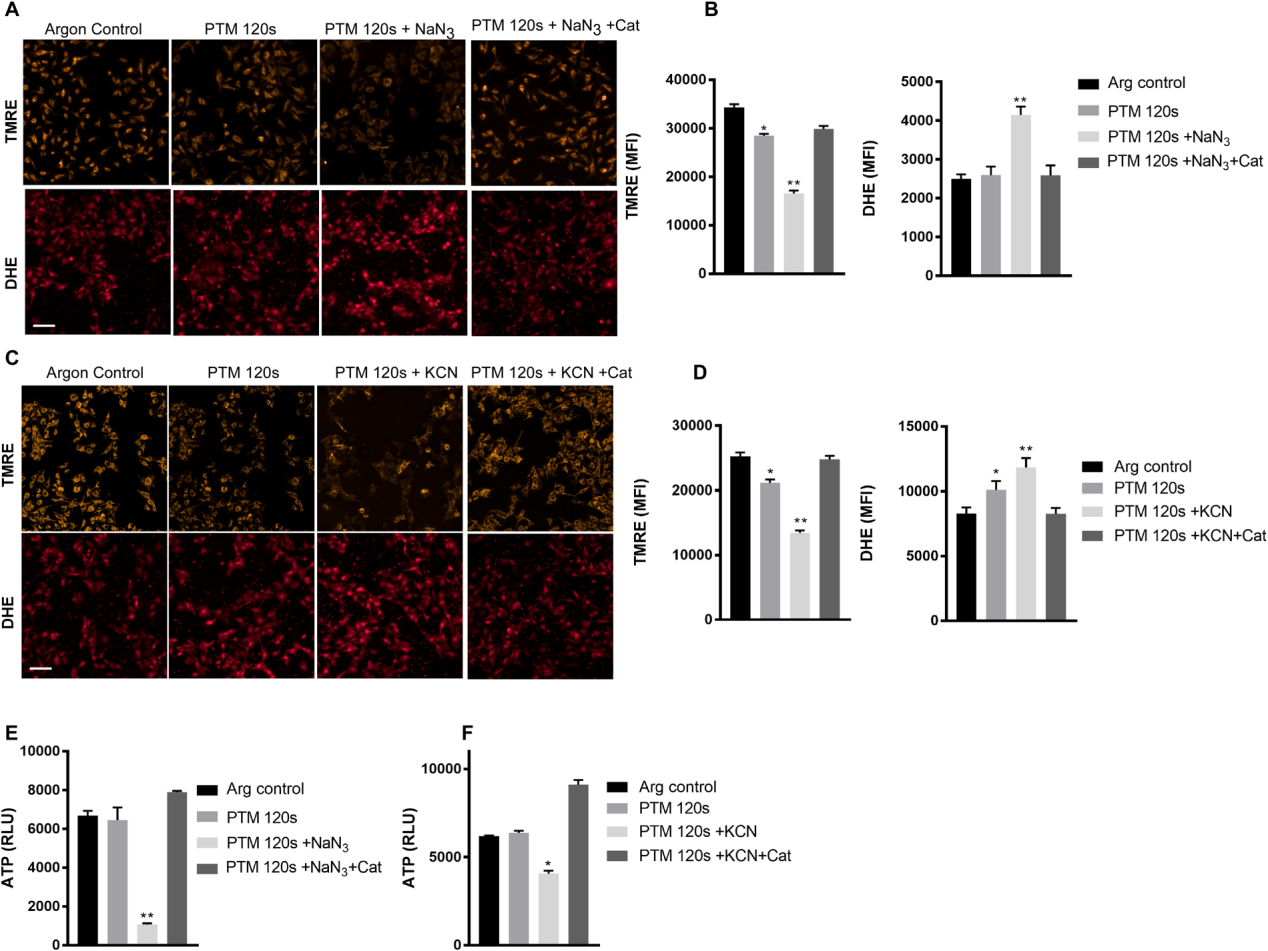
CcO inhibition and PTM induce mitochondrial dysfunction. (**A**) Mitochondrial membrane potential enumerated by TMRE dye uptake and cellular superoxide levels by nuclear DHE staining in B16F10 cells following 3 h treatment with NaN_3_. (**B**) Quantification of fluorescence intensity from (**A**). (**C**) Mitochondrial membrane potential enumerated by TMRE dye uptake and cellular superoxide levels by nuclear DHE staining in B16F10 cells following 3 h treatment with KCN. (**D**) Quantification of fluorescence intensity from (**C**,**E**) Cellular ATP levels following 2 h treatment with NaN_3_ and PTM. (**F**) Cellular ATP levels following 2 h treatment with KCN and PTM. Scale bar: 100 µm Data are mean + SEM from three independent experiments.

To understand the contributory mechanism of the synergistic of cell death, we quantified the mitochondrial membrane potential by TMRE and superoxide levels by DHE staining in B16F10 cells using live cell imaging. We observed no significant increase in superoxide levels at 2 h incubation with cells treated with only PTM. However, there was a pronounced increase in superoxide levels with co-incubation with NaN_3_ (Fig. 3A,B) or KCN (Fig. 3C,D). This was accompanied by a loss of mitochondrial membrane potential, evident by reduced uptake of TMRE, which was abrogated by pretreating cells with catalase (Fig. 3A,D). As expected, this went along with a significant drop in ATP levels at 2 h following co-incubation of NaN_3_ (Fig. 3E) or KCN (Fig. 3F) with PTM. These data imply that mitochondrial CcO inhibition and exogenous oxidants accelerate cell death by inducing mitochondrial dysfunction in melanoma cells.

### Synergistic cell death was independent of caspase 3 or 7 activity

**Figure 4:**
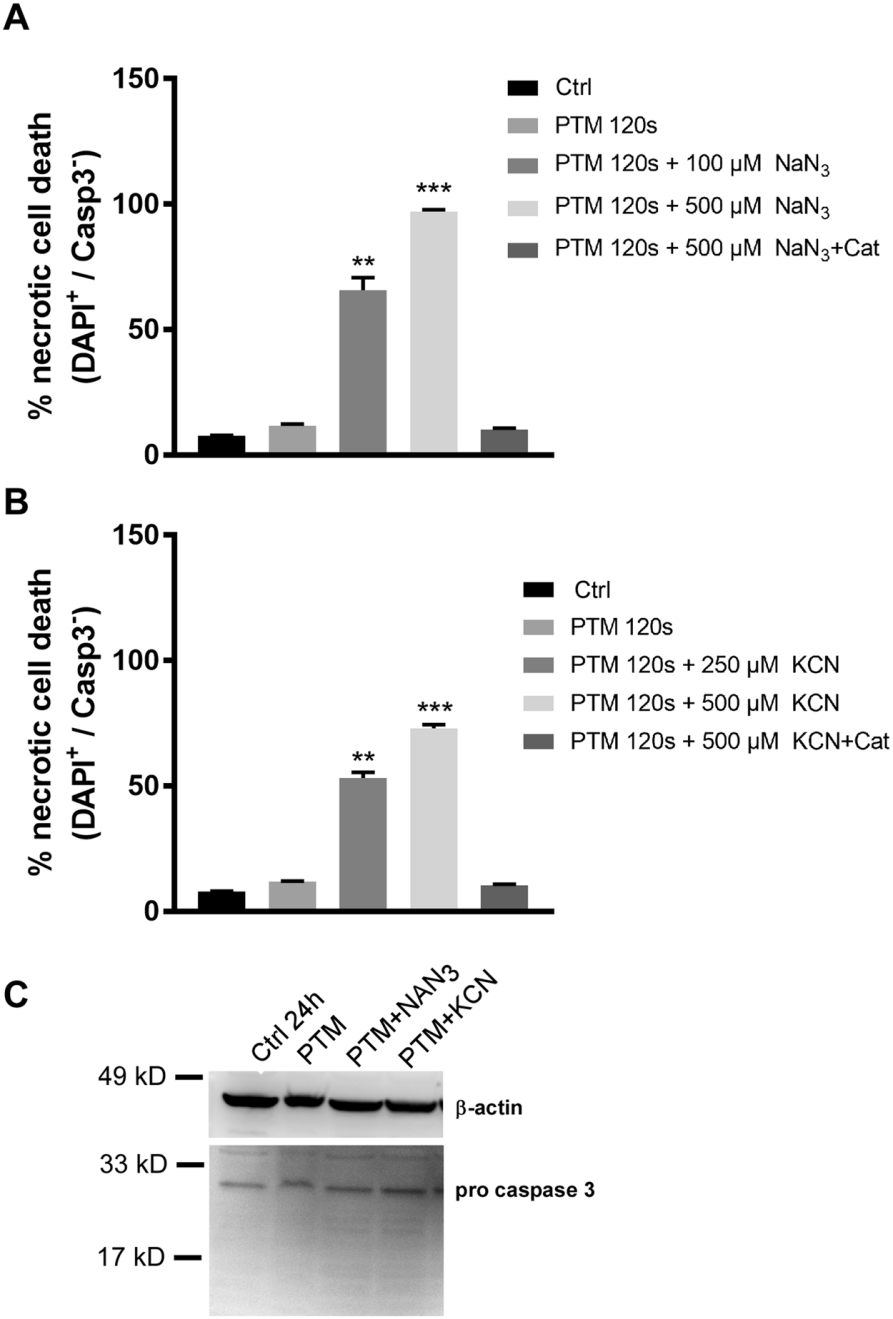
CcO inhibition and PTM induce caspase independent cell death in melanoma cell lines. (**A**,**B**) Flow cytometry analysis of B16F10 cells upon incubation with mitochondrial CcO inhibitors KCN and NaN_3_ in the presence of PTM for 4 h. (**C**) Immunoblotting of whole cell lysates against caspase 3 antibody following treatment of B16F10 cells with mitochondrial CcO inhibitors KCN and NaN_3_ in the presence of PTM for3 h (**A**) Data are mean + SEM from three independent experiments.

Since there was a pronounced mitochondrial dysfunction, we then investigated whether there was an activation of the intrinsic apoptotic pathway preceding cell death in B16F10 cells. Following treatment, cells were incubated with fluorescent indicators for active caspases 3 or 7 and a membrane-impermeable nucleic acid dye to distinguish apoptotic and necrotic populations by flow cytometry (Fig. 4A,B). Results indicated the PTM led to a minor increase in cell death comparable to Fig. 1B. However, induction of cell death was more severe upon co-incubation with KCN or NaN_3_ in a caspase independent mechanism. This was supported by the lack of cleaved caspase 3 at the protein level (Fig. 4C). These data indicate a rapid onset of necrotic cell death in melanoma cells due to acquired energy crisis following incubation with CcO inhibitors and exogenous oxidants.

### SiRNA mediated inhibition of CcO sensitizes melanoma cells to oxidant-induced cell death

**Figure 5:**
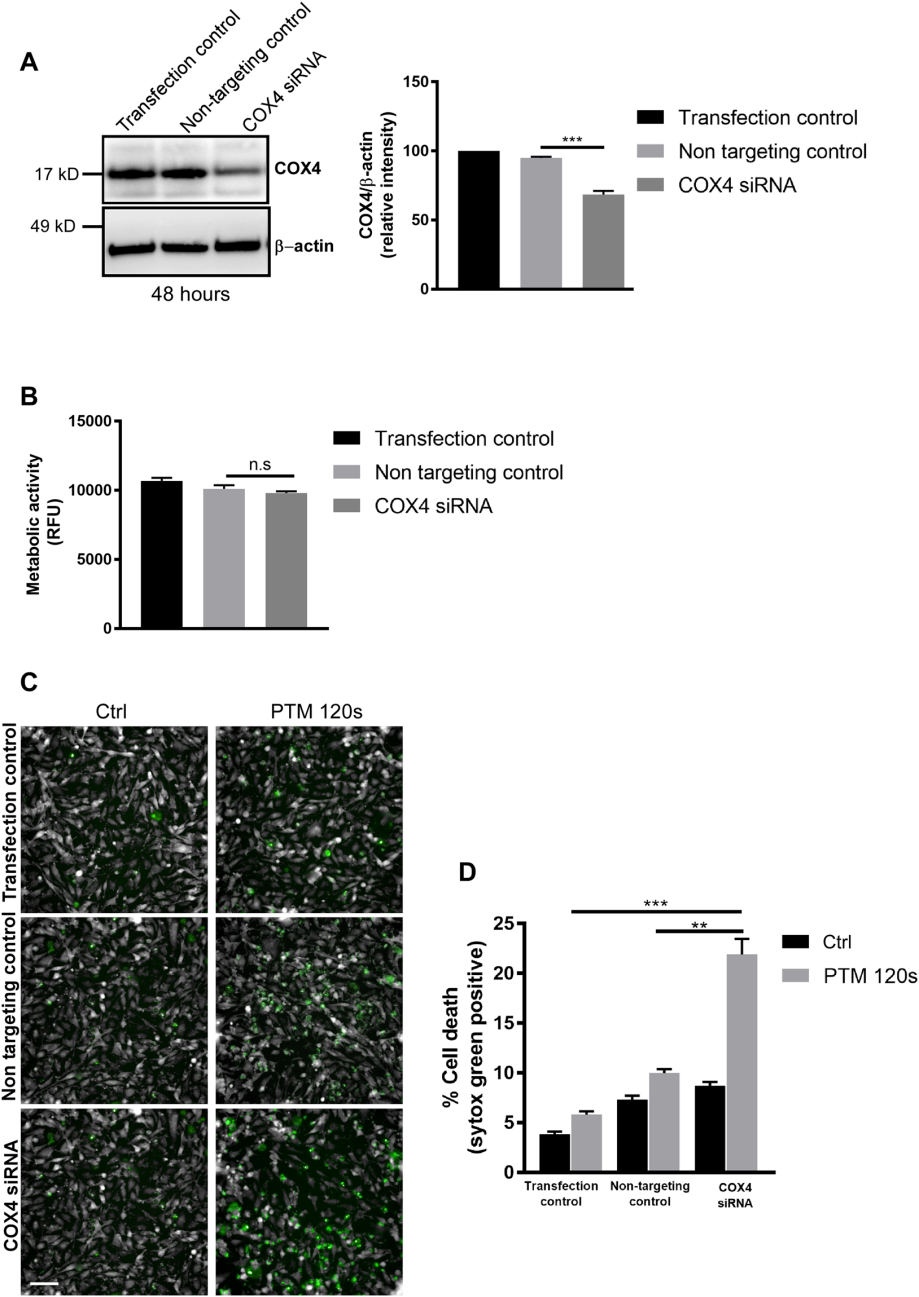
siRNA mediated knockdown of CcO sensitizes SK-MEL-28 cells towards PTM induced cell death. (**A**) Immunoblot and densitometric analysis of SK-MEL-28 cell lysates against COX4 protein following transfection with COX4I1 siRNA with appropriate controls. (**B**) Metabolic activity of SK-MEL-28 cells following COX4I1 siRNA treatment after 48 h. (**C**) Cytotoxicity of SK-MEL-28 cells following CcO knockdown upon incubation with PTM for 6 h. (**D**) Quantification of cell death from (**C**). Data are mean + SEM from three independent experiments.

Biochemical and pharmacological inhibitors are known to have off target effects leading to extrapolated results. Hence to validate our hypothesis we used siRNA based gene knockdown of COX subunit 4 isoform 1 (COX4l1) a major subunit of mitochondrial CcO. We performed knockdown experiments in human SK-MEL-28 cells using siRNA against the COX4l1 with appropriate controls. Silencing CcO was determined at the protein level using anti COX4 antibody and anti β-actin antibody as loading control (Fig. 5A). Cells transfected with COX4l1 siRNA had no significant change in baseline metabolic activity when compared to non-targeting control siRNAs after 24 h (Fig. 5B). However, incubation with PTM led to a significantly higher induction of cell death in COX4 siRNA compared to non-targeting and transfection control cells (Fig. 5C,D). These results validate that inhibition of CcO sensitizes melanoma cells to oxidant induced cell death.

### CcO inhibition by ADDA 5 in the presence of exogenous oxidants leads to melanoma cell death

**Figure 6:**
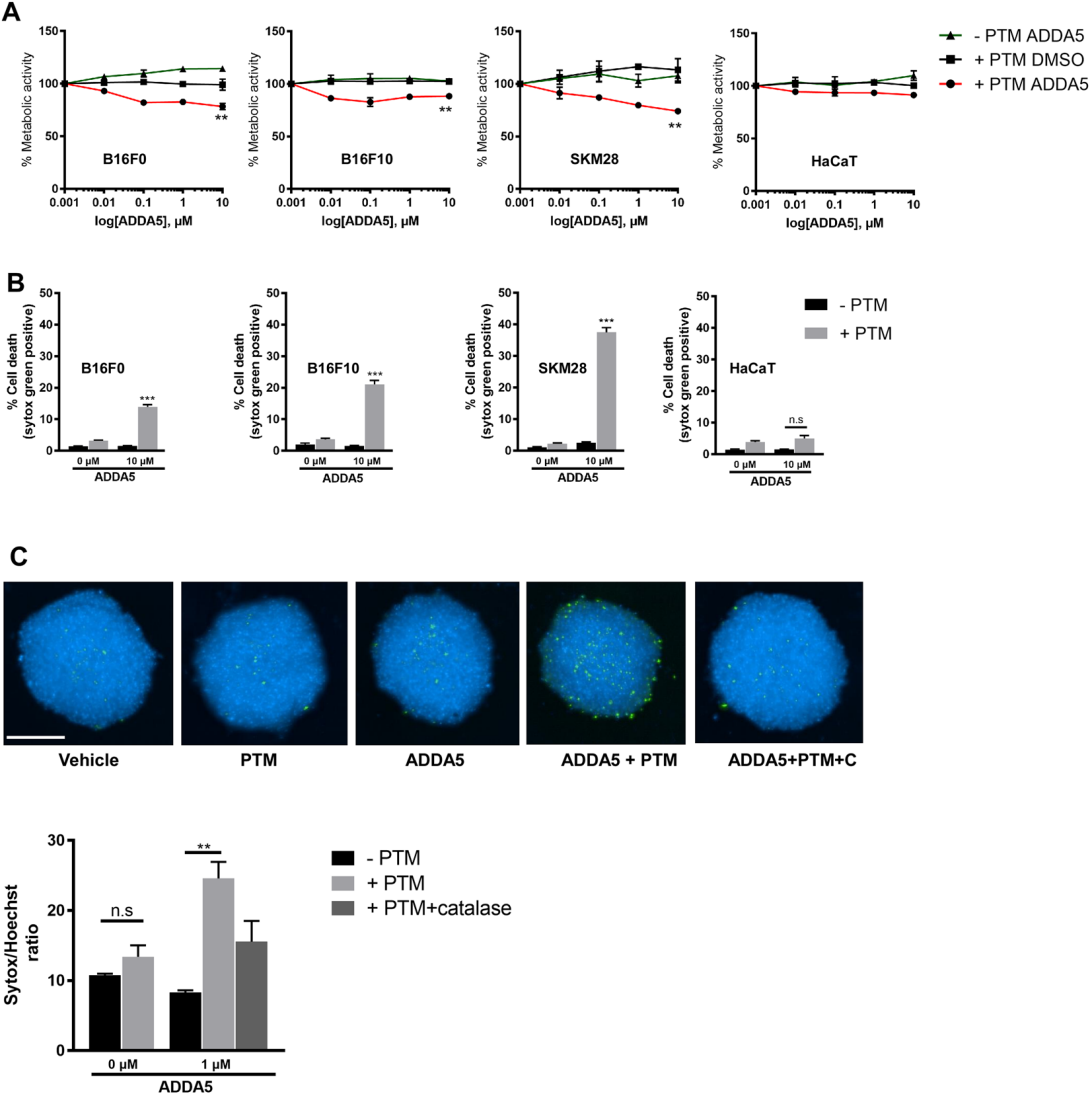
Pharmacological inhibition of CcO using ADDA 5 sensitizes melanoma cells towards PTM induced cell death in 3D spheroids. (**A**) Metabolic activity of melanoma cells incubated with increasing concentrations of ADDA 5 (0.001, 0.01, 0.1 1 and 10 µM) in the presence or absence of PTM for 24 h. DMSO served as vehicle control. (**B**) Cytotoxicity of melanoma cells following ADDA 5 incubation in the presence or absence of PTM for 24 h. (**C**) Representative photomicrographs and quantification of 3D spheroids of SK-MEL-28 cells incubated with 1 µM of ADDA 5 in the presence or absence of PTM for 24 h. Scale bar: 250 µm. Data are mean + SEM from three independent experiments.

ADDA 5 was recently demonstrated to be an effective small molecule inhibitor against CcO activity in glioma cells *in vitro* and *in vivo*. We first determined the cellular metabolic activity in the presence or absence of PTM or ADDA5 over 5 log concentrations during 24 h in all 3 melanoma cell lines and HaCaT. At 10 µM, co-incubation with PTM and ADDA5 resulted in a significantly reduced metabolic activity in melanoma cells but not in non-malignant HaCaT keratinocytes when compared to controls (Fig. 6A). This reduction positively correlated with image quantification of Sytox-positive terminally dead cells after 24 h (Fig. 6B). Finally, we extended our 2D monolayer findings to a 3D tumor spheroid model using SK-MEL-28 cells. Cells within spheroids exhibited terminal cell death upon co-incubation but not monotherapy with ADDA 5 and/or PTM after 24 h at indicated concentrations. The induced toxicity was abrogated by prior addition of catalase (1000 units/ml) indicating exogenous H_2_O_2_ being important to synergize with CcO inhibition in melanoma cell death (Fig. 6C).

## Discussion

We demonstrated that the inhibition of CcO in conjunction with plasma-derived exogenous oxidants might constitute an innovative new treatment approach in melanoma. Consistent with previous studies, we found that melanoma cells are relatively resistant towards oxidative stress^33^. In our study, PTM generating a H_2_O_2_ concentration of 61.3 µM did not have a significant cytotoxic effect on melanoma cells although it was previously shown to decrease their motility^34^. This observation may be attributed to the robust anti-oxidative capacity and pro-oxidant tumor milieu in melanoma cells^11^.

In our study, biochemical inhibition of CcO activity with KCN or NaN3 (≤500 µM) did not alter the viability or metabolic activity of melanoma cells or normal keratinocytes. KCN and NaN3 are routinely used inhibitors of CcO activity in mammalian cells^35^. Previous studies employed higher concentrations (>1 mM) to inhibit CcO activity in multiple cell types^36–38^. However, only few studies have implicated the role of CcO in tumor progression. Genetic knockdown of CcO in human esophageal squamous cell carcinoma led to a shift in metabolic reprogramming and slower growth rates but enhanced tumor invasion^39^. On the contrary, CcO inhibition led to temozolomide-dependent apoptosis in chemoresistant glioma cells suggesting a synergistic role in tumor killing^40^.

Our observed selective and synergistic cytotoxicity of PTM plus CcO inhibition was accompanied by loss of mitochondrial membrane potential and ATP depletion. It has been previously demonstrated that CcO is crucial for stability and activity of mitochondrial complexes I, II, and III^37,41^. CcO inhibition also leads to a subtle increase in superoxide generation at complex I^42^ when the NADH/NAD^+^ ratio is high^43^. Highest superoxide fluxes are seen with reverse electron transport at complex I^44^. Inhibition of complex III leads to elevated superoxide release as well^45^. Consistent with our results, CcO inhibition with, for example, cyanide, is not increasing superoxide production, at least in complex III^46^. Superoxide has a short half-life and is efficiently converted to stable H_2_O_2_ by superoxide dismutase^47^. We found a significant elevation of superoxide only upon CcO inhibition in the presence of PTM, suggesting oxidative stress and subsequent redox signaling as major effector of cellular toxicity. Interestingly, mitochondrial superoxide release is also seen under hypoxic conditions^48^. Connecting to our results, inactivation of hypoxia-inducible factor 1 alpha increases mitochondrial oxidative metabolism, which sensitizes melanoma cells to pro-oxidant killing^49^.

In the current study, there was no activation of executioner caspases 3/7 indicating necrosis as the primary mode of cell death^50^. In general, melanoma cells have been shown to be resistant to apoptotic cell death^51,52^. The absence of Receptor Interacting Protein 3 Kinase (RIP3K) in melanoma cells leads to necroptosis resistance as well^53^. Although the combination treatment led to energy crisis, the observed cell death was restricted to tumor cells. Normal and cancer cells require basal mitochondrial Ca^2+^ uptake for survival, however upon mitochondrial dysfunction and energy crisis, led to activation of autophagy leading cell cycle arrest and survival in normal cells. However, tumor cells bypass cell cycle checkpoints and undergo necrotic cell death during mitosis^54^. Hence, the inability of melanoma cells to slow down proliferation during ATP depletion accounts for their vulnerability to cell killing. Similar finding was recently observed that cold plasma treated medium induced caspase-independent cell death via mitochondrial dysfunction and Ca^2+^ homeostasis in TRAIL resistant human malignant cells^55^.

**Figure 7:**
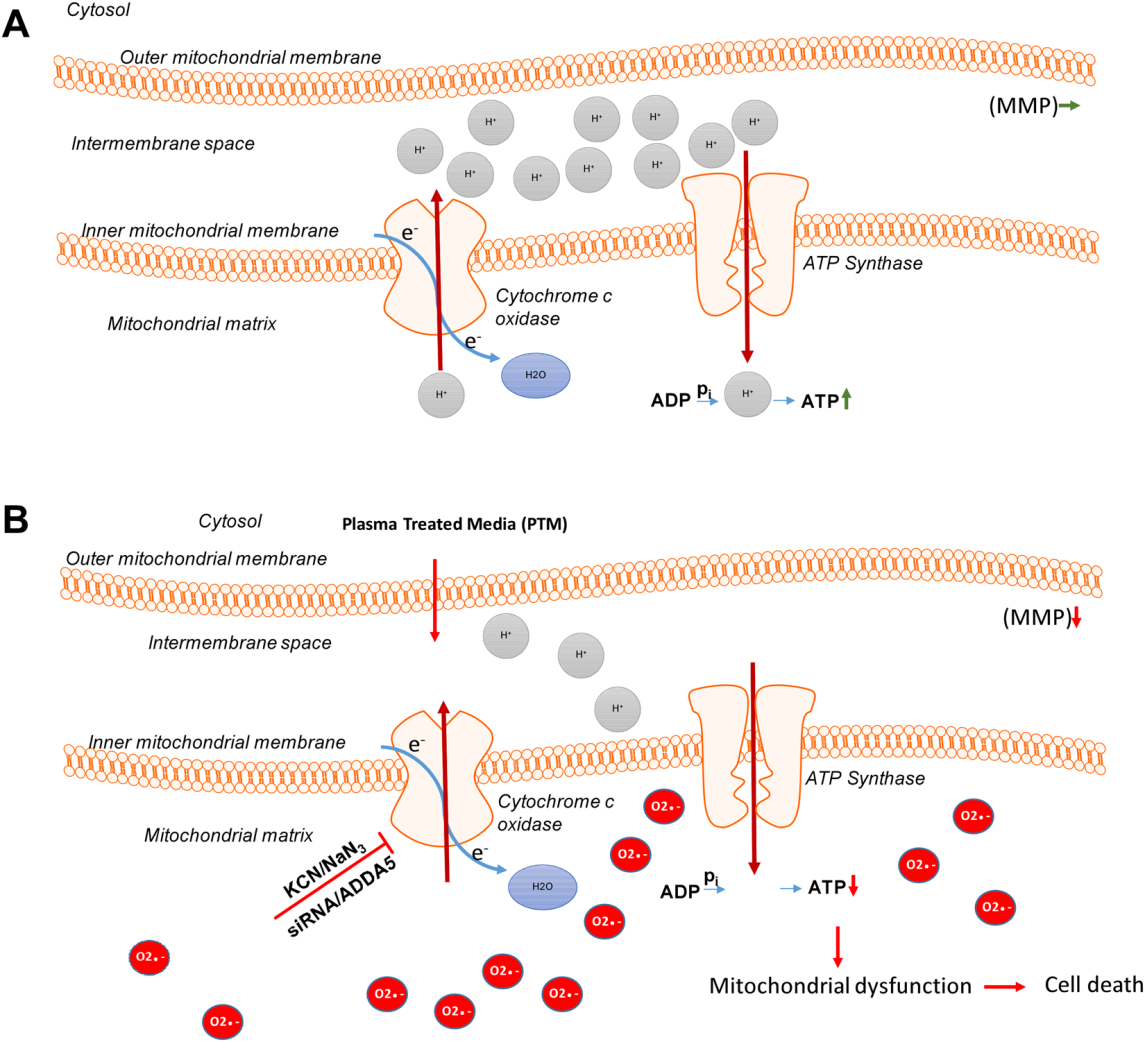
Proposed model for the synergistic effect of CcO inhibition and PTM. (**A**) Melanoma cells exhibit an active oxidative phosphorylation and maintain their mitochondrial membrane potential (MMP) by accumulation of a proton gradient (Grey) and by ATP generation. (**B**) Inhibition of CcO and addition of PTM leads to an increase in superoxide anions (Red) in the mitochondrial matrix that result in loss of MMP and subsequent ATP depletion. This finally leads to energy crisis and cell death.

CcO is a multimeric enzyme composed of several metal prosthetic sites and 14 protein subunits in mammals^49^. We further confirmed our treatment approach (the proposed cellular events are summarized in Fig. 7) by genetically knocking down the subunit complex IVi1 by siRNA in human SK-MEL-28 cells. Knockdown of subunit IVi1 leads to disruption of the assembly and function of CcO in the mitochondria^56^. As expected, siRNA-mediated dysfunction of CcO led to a synergistic induction of melanoma cytotoxicity when exposed to PTM (alone and in the absence of an additional pharmacological CcO inhibitor).

Finally, we validated our above findings using a specific inhibitor of CcO, ADDA 5, which was recently identified from a large compound screening^57^. The potency of the drug is underlined by the absence of side effects in mice that received up to 80 mg/kg body weight for 7 days. Our combinational approach with ADDA5 and PTM was markedly effective in 2D and 3D melanoma cultures. Furthermore, our study demonstrates ADDA5 (10 µM) is a superior complex IV inhibitor in comparison to KCN or NaN_3_ (>250 µM) and could be used for biochemical studies of electron transport chain.

In conclusion, we demonstrated that inhibition of CcO in the presence of exogenous oxidants sensitized melanoma cells towards mitochondrial dysfunction and cell death. Although targeting oxidative phosphorylation pathways in healthy cells would be detrimental, selective mitochondrial complex IV inhibitors would be not. To this end, a novel inhibitor, ADDA 5, showed no toxicity in mice and significant cytotoxicity towards melanoma cells and spheroids in combination with PTM in our study. The kINPen plasma device has already received approval for clinical applications such as wound healing^58,59^ and showed promising results in palliative cancer care^60,61^. We foresee plasma-derived reactive could also be delivered to cutaneous, metastatic, malignant melanoma due its direct accessibility for plasma treatment or plasma-treated liquids for mono or combination therapy. However, further exploration of this innovative approach is warranted in pre-clinical dermato-oncology.

## Electronic supplementary material

Supplementary dataset 1
