# Supplementary material for: Cytochrome C oxidase Inhibition and Cold Plasma-derived Oxidants Synergize in Melanoma Cell Death Induction
Source: Sci Rep. 2018 Aug 24;8:12734. doi: 10.1038/s41598-018-31031-2 (PMC6109085; doi:10.1038/s41598-018-31031-2)

Cytochrome C oxidase Inhibition and Cold Plasma-derived Oxidants Synergize in Melanoma Cell Death Induction

Rajesh Kumar Gandhirajan^1^, Katrin Rödder^1^, Yana Bodnar^1^, Gabriella Pasqual-Melo^1^, Steffen Emmert^2^, Corinne E. Griguer^3^, Klaus‑Dieter Weltmann^1^, Sander Bekeschus^1^

^1^ Leibniz-Institute for Plasma Science and Technology (INP Greifswald), ZIK *plasmatis*, Felix‑Hausdorff‑Str. 2, 17489 Greifswald, Germany

^2^ University Medical Center Rostock, Clinic for Dermatology and Venerology, Strempelstr. 13, 18057 Rostock, Germany

^3^ University of Birmingham, Department of Neurosurgery, 1670 University Boulevard Birmingham, Alabama 35233, USA

**Figure S1. *Mitochondrial CcO inhibitor sodium azide (NaN_3_) selectively decreases H2O2 mediated metabolic activity in SK-MEL-28*** ***cells but not normal HaCaT cells*.** Comparison of metabolic activity of human melanoma cells and normal human keratinocytes following treatment with 50 µM H_2_O_2_ and 500 µM NaN_3_ for 6 h. Data are mean +SEM from three independent experiments.


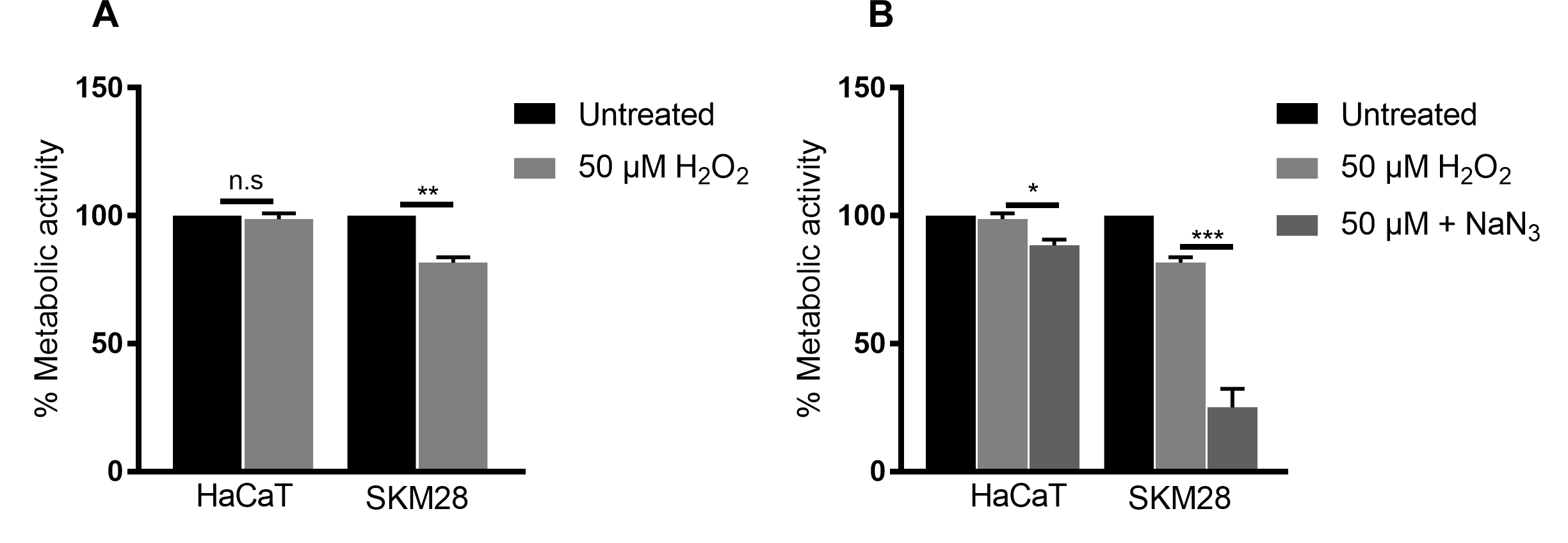


**Figure S2*.*** ***H_2_O_2_ decreases the metabolic activity of melanoma following treatment with KCN or NaN_3_*** (A) Metabolic activity of melanoma cells incubated with indicated concentrations of KCN in the presence 50 µM H_2_O_2_ for 6 h. (B) Metabolic activity of melanoma cells incubated with indicated concentrations of KCN in the presence 50 µM H_2_O_2_ for 6 h. Catalase (1000 IU/ml) served as quencher. Data are mean +SEM from three independent experiments.


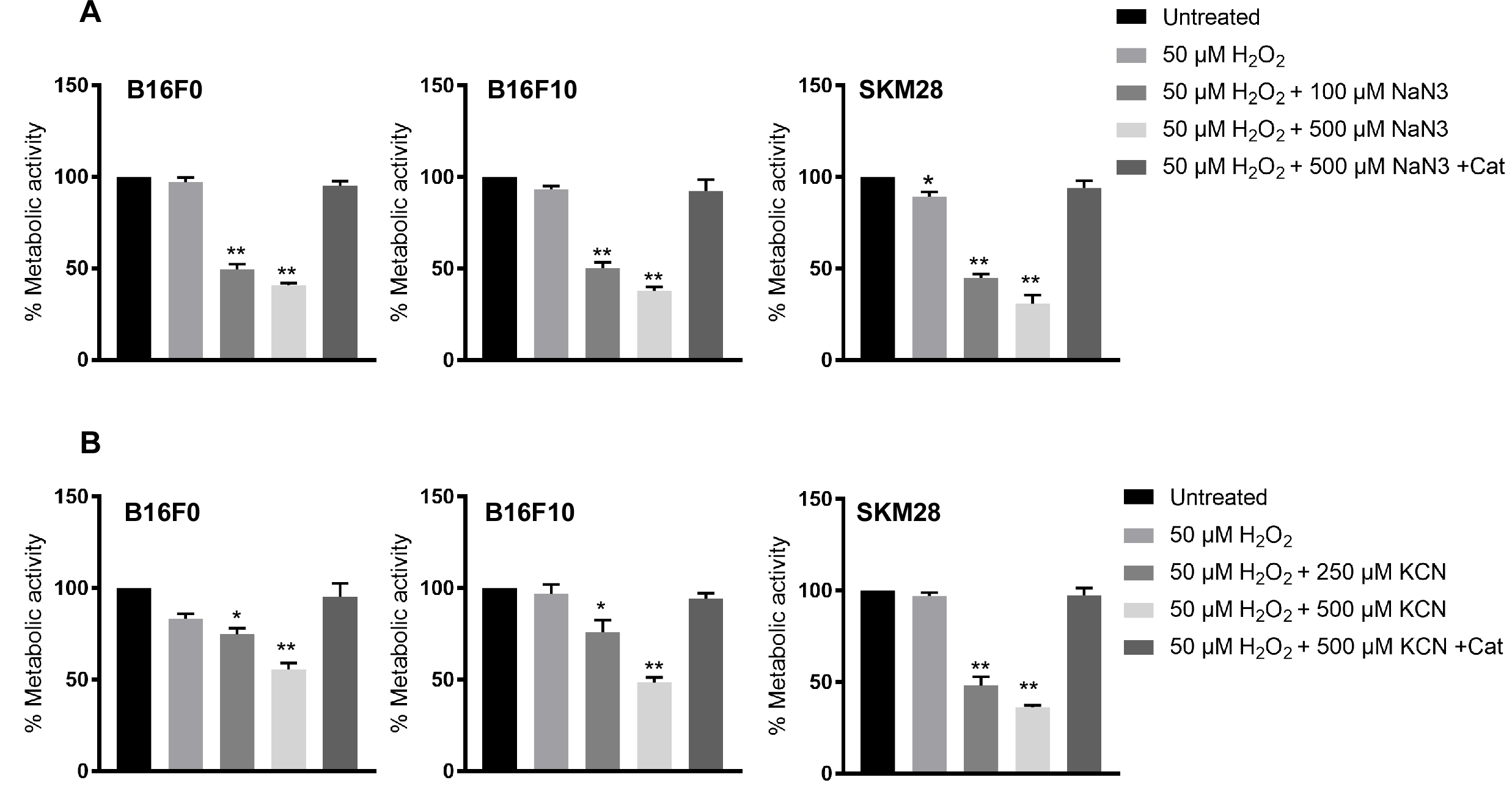

Supplement: Supplementary file 1 — Supplementary dataset 1 [file 41598_2018_31031_MOESM1_ESM.docx]
